# Cat-D: a targeted sequencing method for the simultaneous detection of small DNA mutations and large DNA deletions with flexible boundaries

**DOI:** 10.1038/s41598-017-15764-0

**Published:** 2017-11-16

**Authors:** Ru Hong, Udita Chandola, Li-Feng Zhang

**Affiliations:** School of Biological Sciences, Nanyang Technological University, 60 Nanyang Drive, Singapore, 637551 Singapore

## Abstract

We developed a targeted DNA sequencing method that is capable of detecting a comprehensive panel of DNA mutations including small DNA mutations and large DNA deletions with unknown/flexible boundaries. The method directly identifies the large DNA deletions (Cat-D) without relying on sequencing coverage to make the genotype calls. We performed the method to simultaneously detect 10 small DNA mutations in β-thalassemia and 2 large genomic deletions in α-thalassemia from 10 genomic DNA samples. Cat-D was performed on 8 genomic DNA samples in duplicate. The 18 Cat-D samples were combined in one sequencing run. In total, 216 genotype calls were made, and 215 of the genotype calls were accurate. No false negative genotype calls were made. One false positive genotype call was made on one target mutation in one experimental duplicate from a genomic DNA sample. In summary, Cat-D can be developed into a robust, high-throughput and cost-effective method suitable for population-based carrier screens.

## Introduction

Although deep sequencing technologies have made personal genome sequencing possible, the wide use of the technology on population-based carrier screens for genetic disorders is limited by the lack of a robust and cost-effective targeted sequencing method capable of detecting large DNA deletions.

**Figure 1:**
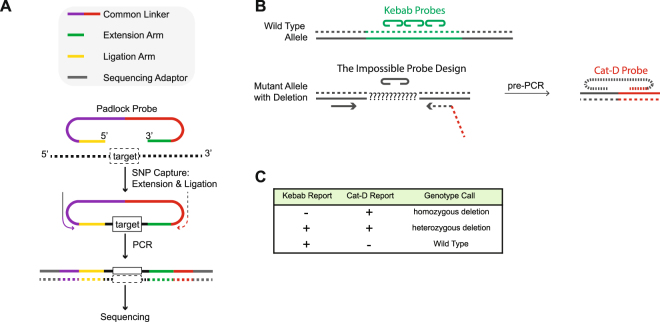
The experimental design of Cat-D. (**A**) A general method for padlock capture. Note: Solid and dashed lines indicate the sense and antisense strands of the DNA templates, respectively. (**B**) The designs of the “Cat-D” and “Kebab” padlock probes. (**C**) Cat-D and Kebab padlock probes are used together in a padlock probe library to make the genotype calls for large genomic DNA deletions.

First, it is important to have a suitable approach to concentrate the sequencing power onto a short list of target DNA regions (targeted sequencing). Without target enrichment, the vast majority of the sequencing power will be wasted on aimlessly sequencing the entire genome (3 billion base pairs). Padlock capture^1,2^ is an available targeted sequencing method. A padlock probe is a DNA oligo designed for a DNA target (Fig. 1A). Each padlock probe carries an extension arm and a ligation arm, which are designed specifically for the DNA target. Similar to a pair of PCR primers, the two arms bind to the template DNA through complementary base pairing, but differently from a PCR primer pair in that they bind to the same strand of a DNA molecule. After the probe binds to its DNA template, the 3′-end of the extension arm primes for DNA polymerase DNA chain elongation. When the elongation reaction reaches the 5′-end of the ligation arm, the padlock can be “locked up” by ligases to form a single-stranded circular DNA molecule. The rest of the linear DNA molecules in the reaction can then be efficiently removed by exonucleases. The common linker sequence of each padlock probe allows a common PCR primer pair to amplify all the padlock capture products for deep sequencing. It has been shown that a padlock probe library containing tens of thousands of padlock probes worked efficiently^1,3^. Compared with other available methods for targeted sequencing, padlock capture is more suitable for population-based carrier screens, as once synthesized, the padlock library can be easily regenerated by PCR, whereby microarrays or RNA baits used for target enrichment in other methods are expensive and non-reusable^4^.

Second, the targeted sequencing method should be able to detect large DNA deletions with flexible or unknown deletion boundaries, as these types of mutations are frequently seen in human genetic disorders. A well-known example is thalassemia, an inherited blood disorder caused by mutated genes encoding the hemoglobin α-chain (α-thalassemia) and β-chain (β-thalassemia)^5^. Hemoglobin defects cause red blood cell malfunctions and result in mild or severe anemia. However, the same defect also provides a degree of protection against malaria. The selective survival advantage of heterozygous carriers is believed to be responsible for perpetuating the mutations in human populations^6^. Thalassemia is one of the most common genetic disorders worldwide, posing an important public health problem in Southeast Asia, the Mediterranean region, the Middle East and sub-Saharan Africa^5^. Approximately 18% of the population in Guangxi province (China)^7^ and 3% of the Singaporean population (https://www.kkh.com.sg/HealthPedia/Pages/PregnancyPlanningForBabyThalassaemia.aspx) are carriers of thalassemia mutations. In contrast to the point mutations commonly seen in β-thalassemia^8,9^, the common mutations found in α-thalassemia are a series of large DNA deletions (~3–40 kb)^10^ (Supplementary Fig. S1). Although the carrier rate for thalassemia mutations is extraordinarily high, a population-based carrier screen is difficult to perform. The experimental techniques being used in clinical labs for detecting large DNA deletions in thalassemia^10^, such as gap-PCR, are low throughput (one test for one patient sample) and not comprehensive (one test for one specific mutation). These techniques are only used for patient DNA diagnosis and are unsuitable for population-based carrier screens. It is worth noting that alternative sequencing approaches, such as Nanopore sequencing^11^ and paired-end long-insert Illumina sequencing^12^, are methods capable of detecting large genomic DNA deletions. However, neither method is a targeted sequencing method. Both methods require a suitable target enrichment step if they are to be used for population-based mutation carrier screens. Moreover, both methods are not suitable for the clinical detection of small DNA mutations. Illumina paired-end sequencing is not cost-efficient, as paired-end sequencing is not necessary for the detection of small DNA mutations. For Nanopore sequencing, its high sequencing error rate^11^ makes it very difficult to apply the method for DNA mutation detection, especially for small DNA mutations.

The strength of padlock capture is to detect small DNA mutations such as SNPs (single-nucleotide polymorphism). It is straight forward to design a padlock probe library targeting a panel of small DNA mutations. However, the panel cannot include thalassemia DNA deletions, which is one of the most common mutations in human genetic disorders. The length of the DNA region captured by a padlock probe is restricted by the length limits of the synthesized padlock probes^13^. For a large DNA deletion with flexible or unknown deletion boundaries, it is difficult and unreliable to design a padlock probe to directly capture the junction region of the deletion (Fig. 1B, the “impossible” design). Alternatively, a series of padlock probes can be designed to cover the deleted region (Fig. 1B, the “Kebab” design). One can imagine that these padlock probes bind to the template DNA and form a “Kebab” shape. Therefore, we named these padlock probes Kebab probes. Kebab probes return negative results from homozygous mutants. However, they cannot help to distinguish heterozygous mutants from the wild type, which is the most important genotyping information for a population-based carrier screen. Taken together, the large genomic deletions observed in thalassemia represent a special type of mutation that is frequently observed in human genetic disorders but is difficult to detect using conventional sequencing approaches.

## Results

### Experimental design of Cat-D

We developed a method of using padlock probes to positively “catch a large deletion” (Fig. 1B, the “Cat-D” design). The method does not rely on a negative readout to “detect” the deletion. It also does not rely on using sequencing data to reveal the “gene copy number variation”. In Cat-D, the first step is a PCR reaction (Fig. 1B, pre-PCR). A pair of PCR primers is designed to amplify the DNA region surrounding the deletion. Because of the flexible PCR amplicon length, designing the PCR primers does not depend on knowing the exact deletion boundaries. Only the mutant allele carrying the large DNA deletion can be amplified. The wild type allele is not PCR-amplified because the deletion size is too large to allow the primer pair to work along the wild type allele. The basic concept of the pre-PCR in Cat-D is the same as a commonly used technique called gap PCR. In contrast to gap PCR, one of the two pre-PCR primers in Cat-D carries an adaptor sequence on its 5′-end (Fig. 1B, labeled in red). The adapter sequence is artificially designed to ensure the sequence does not exist in the human genome. The adaptor complementary strand is produced only if the PCR works. Because padlock capture is strand-specific, a special padlock probe, the “Cat-D probe” (Fig. 1B), can be designed to capture the pre-PCR product with its extension arm targeting the adaptor complementary strand. The Cat-D probe only works if the PCR works. To avoid detecting the noise associated with non-specific primer binding, which may occur during a PCR reaction, the ligation arm of the Cat-D probe is designed to capture the DNA region immediately downstream of the pre-PCR primer. In summary, genotype calls for large deletions can be made by the padlock capture results from Cat-D probes together with Kebab probes (Fig. 1C).

To catch multiple large deletions, multiple primer pairs targeting different deletions can be included in one pre-PCR reaction. Each primer pair targets one deletion and provides one unique adaptor sequence for designing the corresponding Cat-D probe. There is no restriction to the amplicon size of each primer pair. The amplicon sizes of different primer pairs can be similar or different. The pre-PCR product is subjected to the padlock capture of a probe library, which includes Cat-D probes and other padlock probes targeting a comprehensive panel of DNA mutations.

### Pre-PCR cycle optimization and test run setup

**Figure 2:**
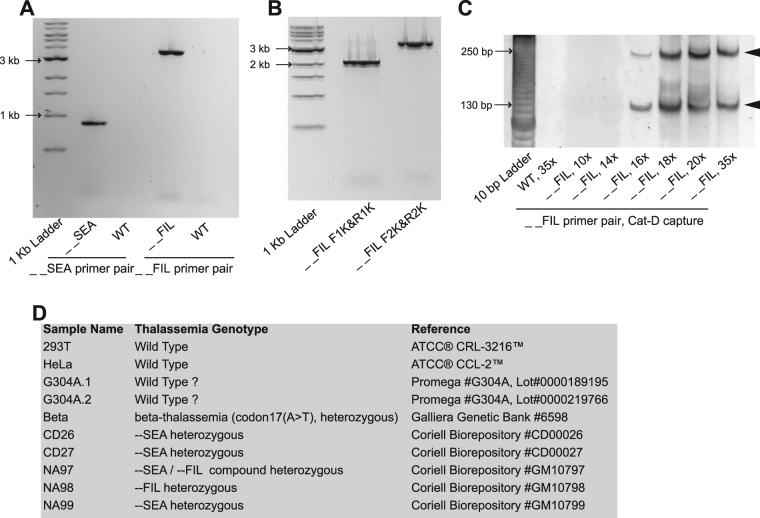
Optimization of pre-PCR cycles and test run setup. (**A**) Gap PCR results for two large DNA deletions in α-thalassemia (--SEA and --FIL). (**B**) A gap PCR result from a patient sample (Coriell Biorepository GM10796) shows that the deletion boundary of --FIL differs among individual patient samples. The PCR amplicon size was estimated according to a previous publication^14^. The predicted PCR amplicon sizes are included in the primer names. (**C**) Padlock capture using Cat-D padlock probes successfully detected --FIL. A PCR primer pair was designed to specifically amplify the Cat-D padlock capture products of --FIL. The orientation of the PCR primers ensures that the primers only amplify the circular DNA template of the successful padlock capture. The arrowheads point to the padlock capture products with the expected sizes. The ~120 bp and ~240 bp bands correspond to PCR extension around the circular DNA templates (a unique feature of successful padlock capture) 1 and 2 times, respectively. Cat-D was successful with a minimum of 16 pre-PCR cycles. The wild type sample returned negative results even after a full 35-cycle pre-PCR. (**D**) Genomic DNA samples used in this study. Note: Uncropped images of the full-length gels shown in this figure are presented in Supplementary Fig. S6.

The pre-PCR product is subjected to padlock capture as a downstream assay. Therefore, the pre-PCR does not have to be completed with full PCR cycles. We first performed gap PCR and successfully detected two thalassemia deletions from patient genomic DNA samples (Fig. 2A). Interestingly, the PCR amplicon sizes from the patient sample (Coriell Biorepository GM10796) were ~1 kb longer than the PCR amplicon sizes estimated based on a previous publication^14^ (Fig. 2B). This result further confirmed that the deletion boundaries vary among patient samples. The number of pre-PCR cycles required for Cat-D was then tested. --FIL was successfully detected by Cat-D with a minimum of 16 pre-PCR cycles (Fig. 2C).

We generated a padlock probe library containing 5 padlock probes targeting the Cat-D product of --FIL, 5 padlock probes targeting the Cat-D product of --SEA, 17 Kebab probes targeting the commonly deleted regions in --FIL and --SEA, and 9 padlock probes targeting 10 different small β-thalassemia DNA mutations.

We performed a test run on a collection of 10 human genomic DNA samples (Fig. 2D). This study was approved by the Ethics Committee of Nanyang Technological University. Padlock capture was performed on each sample in duplicate. Two genomic DNA samples from two commonly used human cancer cell lines (293 T and HeLa) are regarded as “wild type” samples, as the samples were tested as “wild type” for all the thalassemia mutations included in this study (data not shown). Six α-thalassemia genomic DNA samples and one β-thalassemia genomic DNA sample were included. A special human DNA sample was purchased from Promega (Cat# G304A). The sample was originally included in this study as a wild type control. However, we later realized that Promega (Cat# G304A) is prepared from human whole blood from multiple anonymous donors. The blood samples are only tested as negative for HIV and Hepatitis B. There is no information available regarding the samples’ genotypes for thalassemia mutations. Therefore, G304A should be regarded as a special DNA sample without a clear genotype. We included G304A in this study just for the test run. Moreover, our padlock capture duplicates on the sample (G304A.1 and G304A.2) were performed on G304A from two different lots (G304A.1 LOT0000189195; G304A.2 LOT0000219766). Therefore, G304A.1 and G304A.2 should be considered two different DNA samples.

On average, ~184 K reads were obtained from each sample. To confirm the experimental consistency of the method, we calculated the correlation coefficients between the duplicates in each sample. The correlation coefficient of the eight experimental duplicates was 0.98 ± 0.01 (Supplementary Fig. S2). This result confirmed the high experimental consistency of the method.

### Large α-thalassemia DNA deletions detected by Cat-D

**Figure 3:**
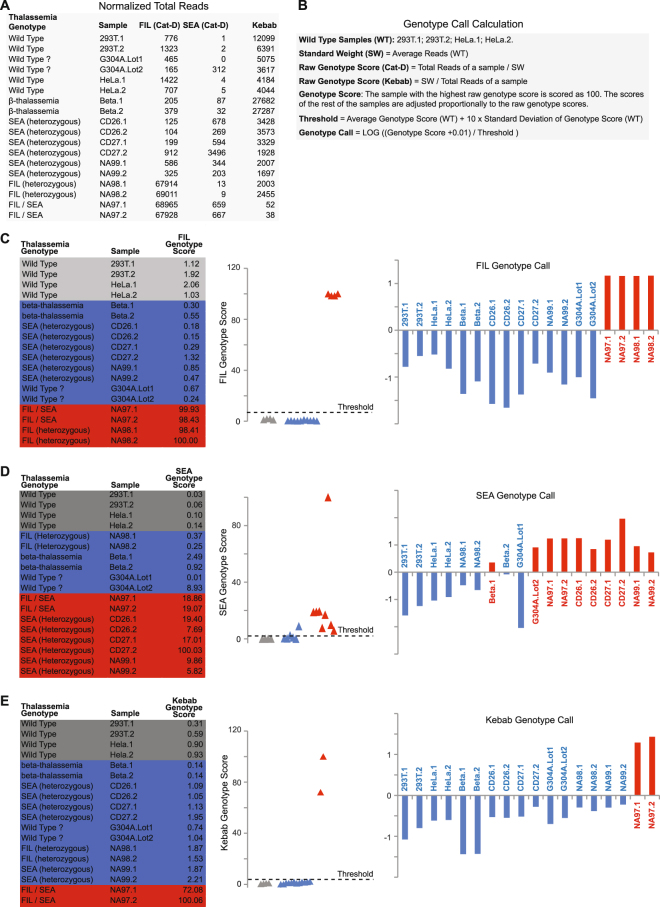
Genotype scores and genotype calls for α-thalassemia mutations. (**A**) Sequencing data headcounts. For each sample, the total count of all the mapped reads from all the Cat-D probes targeting --FIL is taken as the headcount for --FIL (Cat-D). The same analysis was performed to generate the headcounts for --SEA (Cat-D) and Kebab. The sequencing depth was normalized to 200 K reads per sample. (**B**) The mathematical method to calculate the genotype scores and to make the genotype calls on large DNA deletions detected by Cat-D probes and Kebab probes. (**C**) --FIL. (**D**) --SEA. (**E**) Kebab. Note: For genotype scores, the samples are labeled in gray (wild type), red (mutants) and blue (genotypes to be tested). For genotype calls, samples are labeled in red (positive genotype calls) and blue (negative genotype calls).

The raw data (Fig. 3A) clearly showed that the padlock capture products from the Cat-D probes are significantly higher in the samples carrying the corresponding deletions. The headcounts of the Kebab probe capture products are also significantly lower in the samples containing the compound heterozygous deletion (--FIL/--SEA).

To provide a mathematical justification and generate a computational method to make genotype calls, we established a mathematical method to calculate the genotype scores and make the genotype calls for each sample (Fig. 3B; Methods). The results are nearly picture-perfect for --FIL and Kebab (Fig. 3C,E). Negative genotype calls were accurately made on all the wild type samples and the samples expected to be wild type; for example, the β-thalassemia samples (Beta.1 and Beta.2) are expected to be wild type for the α-thalassemia mutations. Positive genotype calls were also accurately made on all the mutant samples. Clear genotype calls were also made for --SEA (Fig. 3D). All the mutant and wild type samples were accurately genotyped. For the samples “expected” to be wild type, G304A.Lot2 and Beta.1 were genotyped as positive for --SEA (Fig. 3D). G304A is a mixture of genomic DNA isolated from multiple donors, and no information is available regarding the sample’s genotype regarding thalassemia mutations. Based on our genotyping results, it is highly likely that one or more G304A.Lot2 donors are carriers of --SEA. We further confirmed this conclusion by gap PCR (Supplementary Fig. S3). Interestingly, all the genomic DNA samples were subjected to gap PCR before Cat-D to confirm the samples’ genotype for the α-thalassemia mutations (Supplementary Fig. S3A). Each PCR, which contained 100 ng of genomic DNA, was performed for 35 cycles. --SEA was not detected in G304A.Lot2. When gap PCR was repeated with 38 cycles and 200 ng genomic DNA, a clear PCR product for --SEA was detected in G304A.Lot2. This result confirmed the Cat-D genotyping results and showed that Cat-D is more sensitive than gap PCR. For Beta.1, the genotype call is a false positive result. This false positive result can be dealt with by comparing it with the genotype call made on the duplicate sample (Beta.2).

### β-thalassemia point mutations detected by padlock probes

**Figure 4:**
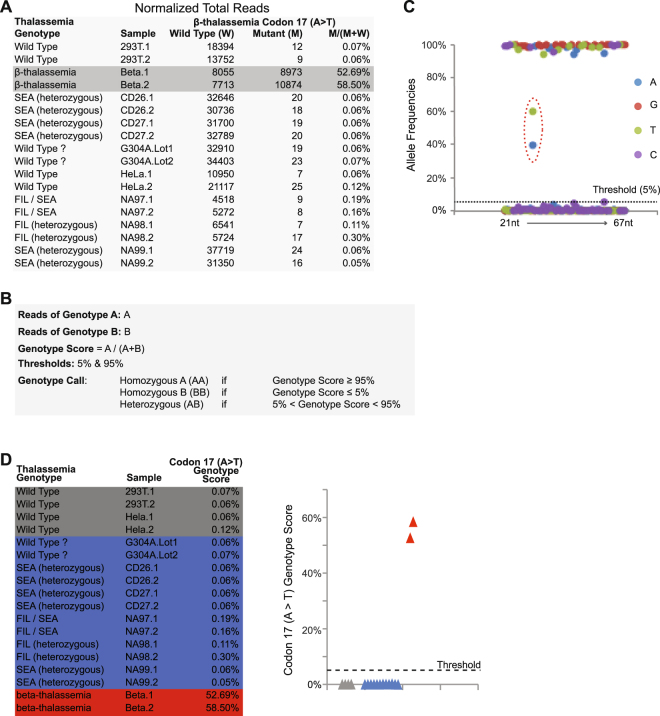
Genotype scores and calls for the β-thalassemia point mutation. (**A**) Sequencing data headcounts. (**B**) The mathematical method to calculate the genotype scores and to make the genotype calls on SNPs and other small DNA mutations. (**C**) Allele frequencies of the padlock capture products. To determine the minor allele frequency used in the data analysis, we calculated the allele frequencies of all the nucleotide positions captured by one padlock probe. The first 20 nucleotides of each sequencing read belong to the ligation arm. The padlock captured region is located between the 21^st^ nucleotide and the 67^th^ nucleotide. For each nucleotide position, we calculated the allele frequency of A, T, C and G. Five percent was selected as the threshold for the minor allele frequency in the data analysis. The position of the β-thalassemia point mutation, codon 17 (A > T), is marked by the red circle. (**D**) Genotype scores.

The Cat-D and Kebab probes only occupy a small fraction of the padlock probe library, which also includes other padlock probes targeting small DNA mutations, such as SNPs. In this study, we included padlock probes targeting small β-thalassemia DNA mutations. One of the 10 DNA samples included in this study is a heterozygous mutant in β-thalassemia codon 17 (A > T). The raw data (Fig. 4A) clearly showed that the mutant headcounts are significantly higher in the samples carrying the corresponding mutation. To provide a mathematical justification and to generate a computational method to make the genotype calls, we established a mathematical method to calculate the genotype call (Fig. 4B). In this case, we simply choose 5% as the threshold to make the genotype call for a “minor allele” (Fig. 4B; Methods). The 5% minor allele frequency was determined by analyzing the padlock capture data (Fig. 4C). We calculated the genotype scores and made the genotype calls on all the samples (Fig. 4D). The results show that the method is sensitive and precise for β-thalassemia point mutations. We also included padlock probes targeting other β-thalassemia small mutations in the padlock probe library. Because we do not have mutant genomic DNA samples for these mutations, we expected that all the samples included in this study are wild type for these mutations. Our genotyping results clearly confirmed our expectations (Supplementary Figs S4 and S5).

## Discussion

In summary, the test run yielded highly satisfying results and a strong proof of concept for Cat-D. These results demonstrate that the method is sensitive (0% false negative rate) and precise (very low false positive rate, ~5% for --SEA mutation). From a clinical point of view, a low false positive rate is more “acceptable” than a low false negative rate. When genetic testing is performed on a large population, the majority of the samples are wild type. With a 0% false negative rate, all the wild type samples can be accurately genotyped and patients can be informed of their testing results with confidence. Regardless of the false positive rate of the experimental method, for the minority of the samples that tested positive for a certain mutation, this is a feasible approach for clinical labs to experimentally validate testing results before “bad news” is released to patients. Taken together, Cat-D is a comprehensive (a single test covers a comprehensive panel of genetic disorders) and high-throughput (one sequencing run contains multiple samples) method suitable for population-based carrier screens.

## Methods

### Primers design

The primer portion of the pre-PCR primers was designed according to the criteria for designing a regular PCR primer. The primers do not bind to repetitive DNA regions in the genome. The primer pairs were confirmed to be able to amplify the target DNA region using a mutant genomic DNA sample carrying the corresponding deletion. For each pre-PCR primer pair, one of the two primers carries the Cat-D adapter on its 5′-portion. The adaptor sequence does exist in the human genome. The adapter sequence was designed to be at least 20 nt in length to achieve sequence specificity and to allow for the design of multiple Cat-D padlock probes. The primers used in the study are listed below:

SEA850F-ADAPTOR (5′-CGATCGTGCGACGCGTATCGGTCCCTTCACCCTCCCACAGTTCCTGC-3′); SEA1KR (5′-TTTCACCCAGTACAGCGAGTCCTTCC-3′); FIL2KR-ADAPTOR (5′-TATGCGTCGCGTGTCGCGCGTAGATCTGCACCTCTGGGTAGGTTC-3′); FIL2KF (5′-TCTCAGGCATGGAAGAATGAGGGC-3′); FILF1K (5′-GAGTTGTAAGATATTTTGGGCCAAGCACG-3′); FILR1K (5′-CTAGAACGTGGATCCAAGAGGGG-3′); and FILR2K (5′-GATCTGCACCTCTGGGTAGGTTC-3′).

### Padlock probe library design

The two arms of each padlock probe were 20 nt or longer. The T_m_ of each arm was optimized to be close to 55 °C. The possibility of each padlock capture target forming complicated secondary structures was minimized using UNAFold (http://homepages.rpi.edu/~zukerm/download/UNAFold_download.html). For each Cat-D padlock probe, the extension arm binds to the complementary sequence of the Cat-D adapter. The ligation arm carries the same DNA sequence as the primer extension product of the pre-PCR primer carrying the Cat-D adapter and is located closely downstream to the 3′-end of the pre-PCR primer carrying the Cat-D adapter.

### Pre-PCR

The Herculase II Fusion DNA Polymerases kit (Cat#600675, Agilent) and 100 ng genomic DNA was used in a 25 µl PCR reaction containing 0.8 µM of each PCR primer and amplified according to the following the PCR program: (1) 95 °C for 3 min; (2) 18 to 20 cycles of 95 °C for 30 sec, 63 °C for 30 sec, and 68 °C for 90 sec; (3) 68 °C for 5 min and (4) a 4 °C hold. The pre-PCR products were purified with the QIAquick PCR Purification Kit (Cat#28104, QIAGEN) and eluted into a 25 µl volume.

### Padlock capture

Padlock capture was performed as previously described^1,3,15^. Briefly, each reaction was performed in 20 µl volume containing 1 unit Ampligase (A3210K, Epicentre), 1 unit Phusion High-Fidelity DNA Polymerase (M0530, New England BioLabs), 1 x Phusion High-Fidelity DNA Polymerase buffer, 10 nM dNTP and 1 ng padlock probe. Two microliters of the purified pre-PCR product and 800 ng genomic DNA were used in each reaction. Nicotinamide adenine dinucleotide (NAD+) was provided in each reaction at a final concentration of 0.5 mM.

### Illumina sequencing

The sequencing libraries were PCR-amplified in a real-time PCR system (CFX Connect, Bio-Rad) using the following primers: (1) CA2-RA.MiSecret (5′-AATGATACGGCGACCACCGAGATCTACACGCTACACGCCTATCGGGAAGCTGAAG-3′); (2) CA-2-FA.Indx3Sol (5′-CAAGCAGAAGACGGCATACGAGATGCCTAACGGTCTGCCATCCGACGGTAGTGT-3′); (3) CA-2-FA.Indx4Sol (5′-CAAGCAGAAGACGGCATACGAGATTGGTCACGGTCTGCCATCCGACGGTAGTGT-3′); (4) CA-2-FA.Indx5Sol (5′-CAAGCAGAAGACGGCATACGAGATCACTGTCGGTCTGCCATCCGACGGTAGTGT-3′); (5) CA-2-FA.Indx7Sol (5′-CAAGCAGAAGACGGCATACGAGATGATCTGCGGTCTGCCATCCGACGGTAGTGT-3′); (6) CA-2-FA.Indx10Sol (5′-CAAGCAGAAGACGGCATACGAGATAAGCTACGGTCTGCCATCCGACGGTAGTGT-3′); (7) CA-2-FA.Indx12Sol (5′-CAAGCAGAAGACGGCATACGAGATTACAAGCGGTCTGCCATCCGACGGTAGTGT-3′); (8) CA-2-FA.Indx13Sol (5′-CAAGCAGAAGACGGCATACGAGATTTGACTCGGTCTGCCATCCGACGGTAGTGT -3′); (9) CA-2-FA.Indx14Sol (5′-CAAGCAGAAGACGGCATACGAGATGGAACTCGGTCTGCCATCCGACGGTAGTGT-3′); (10) CA-2-FA.Indx15Sol (5′-CAAGCAGAAGACGGCATACGAGATTGACATCGGTCTGCCATCCGACGGTAGTGT-3′); (11) CA-2-FA.Indx16Sol (5′-CAAGCAGAAGACGGCATACGAGATGGACGGCGGTCTGCCATCCGACGGTAGTGT-3′); (12) CA-2-FA.Indx18Sol (5′-CAAGCAGAAGACGGCATACGAGATGCGGACCGGTCTGCCATCCGACGGTAGTGT-3′); (13) CA-2-FA.Indx19Sol (5′-CAAGCAGAAGACGGCATACGAGATTTTCACCGGTCTGCCATCCGACGGTAGTGT-3′);(14) CA-2-FA.Indx25Sol (5′-CAAGCAGAAGACGGCATACGAGATATCAGTCGGTCTGCCATCCGACGGTAGTGT-3′); (15) CA-2-FA.Indx45Sol (5′-CAAGCAGAAGACGGCATACGAGATCGTAGTCGGTCTGCCATCCGACGGTAGTGT-3′); (16) CA-2-FA.Indx76Sol (5′-CAAGCAGAAGACGGCATACGAGATAATAGGCGGTCTGCCATCCGACGGTAGTGT-3′); (17) CA-2-FA.Indx91Sol (5′-CAAGCAGAAGACGGCATACGAGATACATCGCGGTCTGCCATCCGACGGTAGTGT-3′); (18) CA-2-FA.Indx92Sol (5′-CAAGCAGAAGACGGCATACGAGATTCAAGTCGGTCTGCCATCCGACGGTAGTGT-3′); and (19) CA-2-FA.Indx93Sol (5′-CAAGCAGAAGACGGCATACGAGATATTGGCCGGTCTGCCATCCGACGGTAGTGT-3′). Each padlock capture product was assigned a unique barcode. The sequencing libraries for each sample were combined. The following sequencing primers were used: (1) Read1.Misecret (5′-ACACGCTACACGCCTATCGGGAAGCTGAAG-3′) and (2) IndexRead (5′-ACACTACCGTCGGATGGCAGACCG-3′). Sequencing was performed on an Illumina MiSeq system using the MiSeq Micro flow cell (2 × 150 cycles). FASTQ files were generated from the sequencer’s output using the Illumina bcl2fastq2 software (v.2.17.1.14) with the default chastity filter set to select the sequence reads for the subsequent analysis.

### Data analysis

We wrote a perl script to find the exact match between the first 88 nt of a sequencing read and an expected padlock probe capture product. To make the genotype calls on large DNA deletions using the padlock capture data from the Cat-D and Kebab probes, a “standard weight” was calculated for each mutation by taking the average headcounts from the four wild type samples (293 T.1, 293 T.2, HeLa.1 and HeLa.2). The raw genotype score of each sample was then calculated as the headcount of the sample divided by the standard weight. Because Kebab probes “negatively” report the corresponding mutation (homozygous deletion), low headcounts indicate the detection of a mutation. Therefore, the raw genotype scores of the Kebab probes were calculated in reverse (standard weight divided by the headcount of each sample). To make the genotype scores more sensible for interpretation, the sample with the highest raw genotype score in the panel was scored as 100. The rest of the samples were scored proportionally to the raw genotype scores. The threshold was then calculated (Fig. 3B). A sample with a genotype score higher than the threshold was positive for the corresponding mutation. The corresponding mutation with the Cat-D probes is a corresponding large DNA deletion. The corresponding mutation with the Kebab probes is a “homozygous” large DNA deletion.

To make the genotype calls on the point mutations, we used 5% as the threshold to make the genotype call on a “minor allele” (Fig. 4B).

## Electronic supplementary material

Supplementary Materials
