# Supplementary material for: Cat-D: a targeted sequencing method for the simultaneous detection of small DNA mutations and large DNA deletions with flexible boundaries
Source: Sci Rep. 2017 Nov 16;7:15701. doi: 10.1038/s41598-017-15764-0 (PMC5691158; doi:10.1038/s41598-017-15764-0)
Supplement: Supplementary file 1 — Supplementary Materials [file 41598_2017_15764_MOESM1_ESM.doc]

**Cat-D: a targeted sequencing method for detecting large DNA deletions with flexible boundaries**

Ru Hong^1^, Udita Chandola^1^ and Li-Feng Zhang^1,*^

^1^School of Biological Sciences, Nanyang Technological University, 60 Nanyang Drive, Singapore 637551.

*Correspondence: zhanglf@ntu.edu.sg


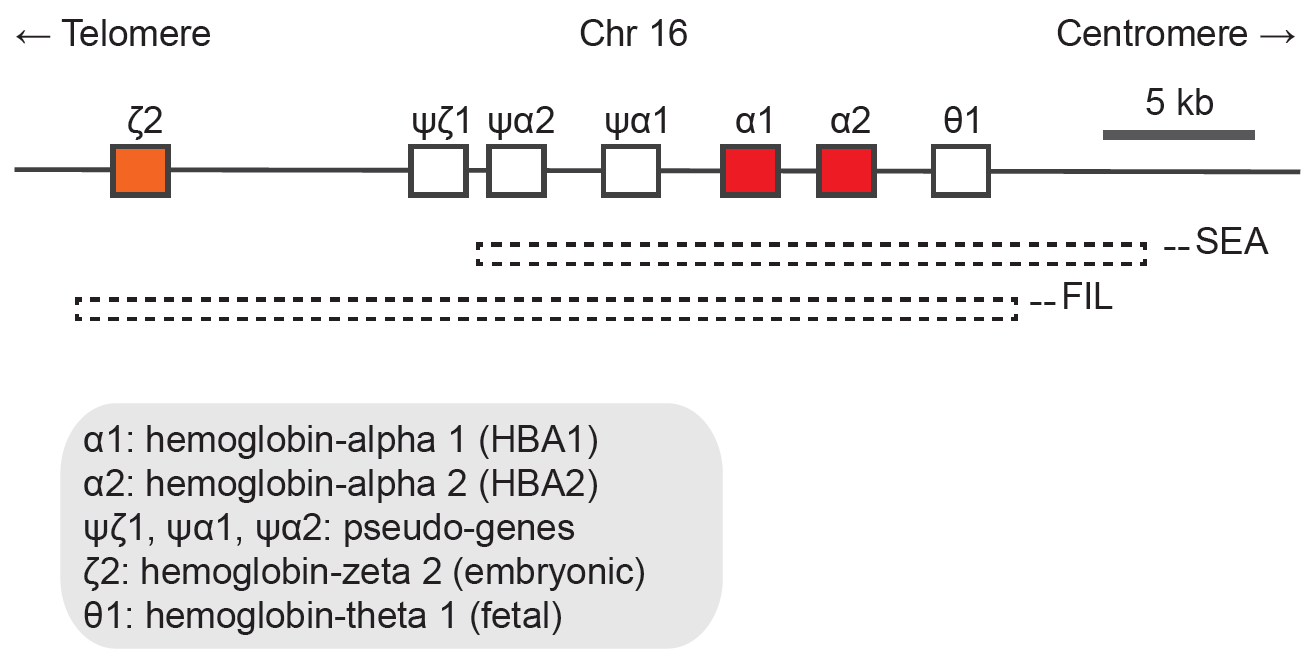


**Supplementary Figure S1.** --FIL and --SEA, two α-thalassemia deletions mainly seen in Southeast Asia.


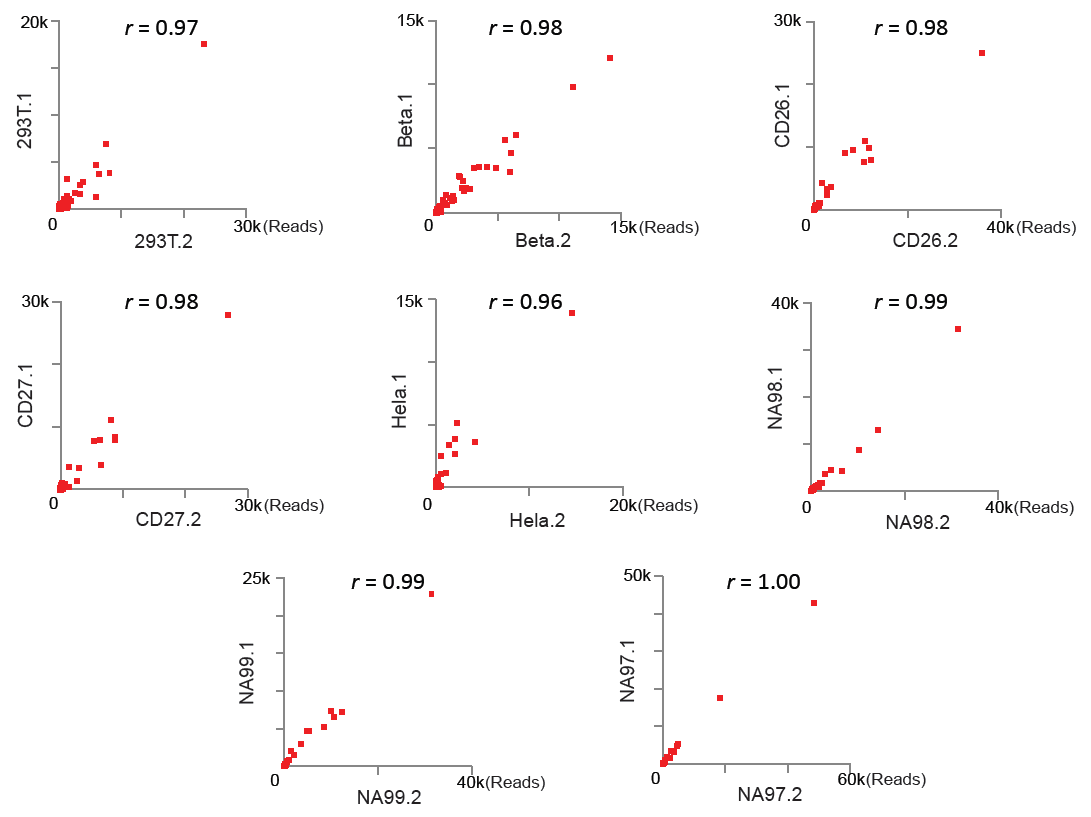


**Supplementary Figure S2**. Correlation co-efficient between padlock capture duplicates of 8 DNA samples. The sequencing depth was normalized to 200K reads per sample. The sequence read counts of each padlock probe in the experimental duplicate are plotted along the x and y axis.


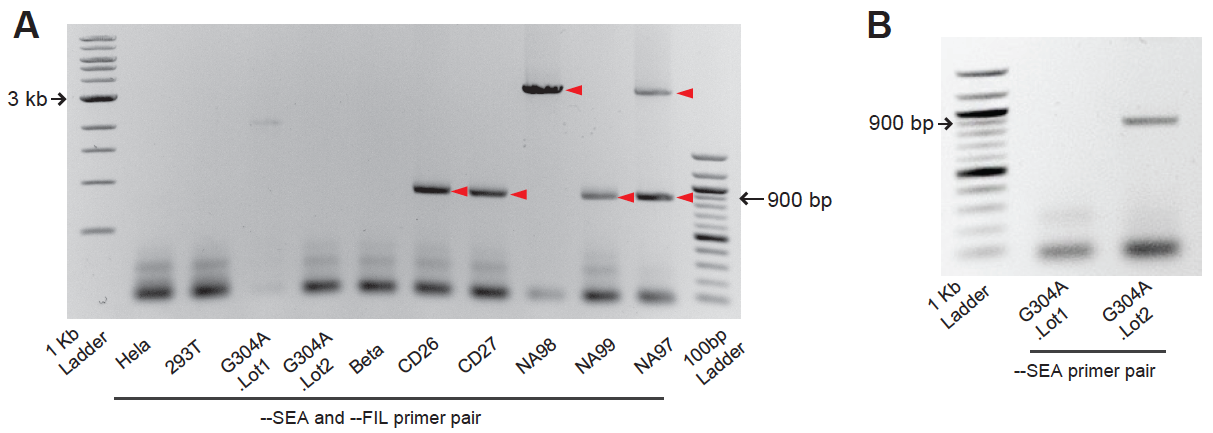


**Supplementary Figure S3.** Gap PCR to detect --FIL and --SEA. (**A**) Each PCR reaction, containing 100 ng genomic DNA, was carried out in 35 cycles. The red arrow heads indicate PCR products with expected size for --FIL (~3 kb) and for--SEA (~900 bp). (**B**) Gap PCR was repeated on G304A.Lot1 and G304A.Lot2. Each PCR reaction, containing 200 ng genomic DNA, was carried out in 38 cycles. A clear PCR product of --SEA was detected in G304A.Lot2. This result confirms the genotyping result of Cat-D and shows that Cat-D is more sensitive than gap PCR. Full-length gels shown in this figure are presented in Supplementary Figure S6.


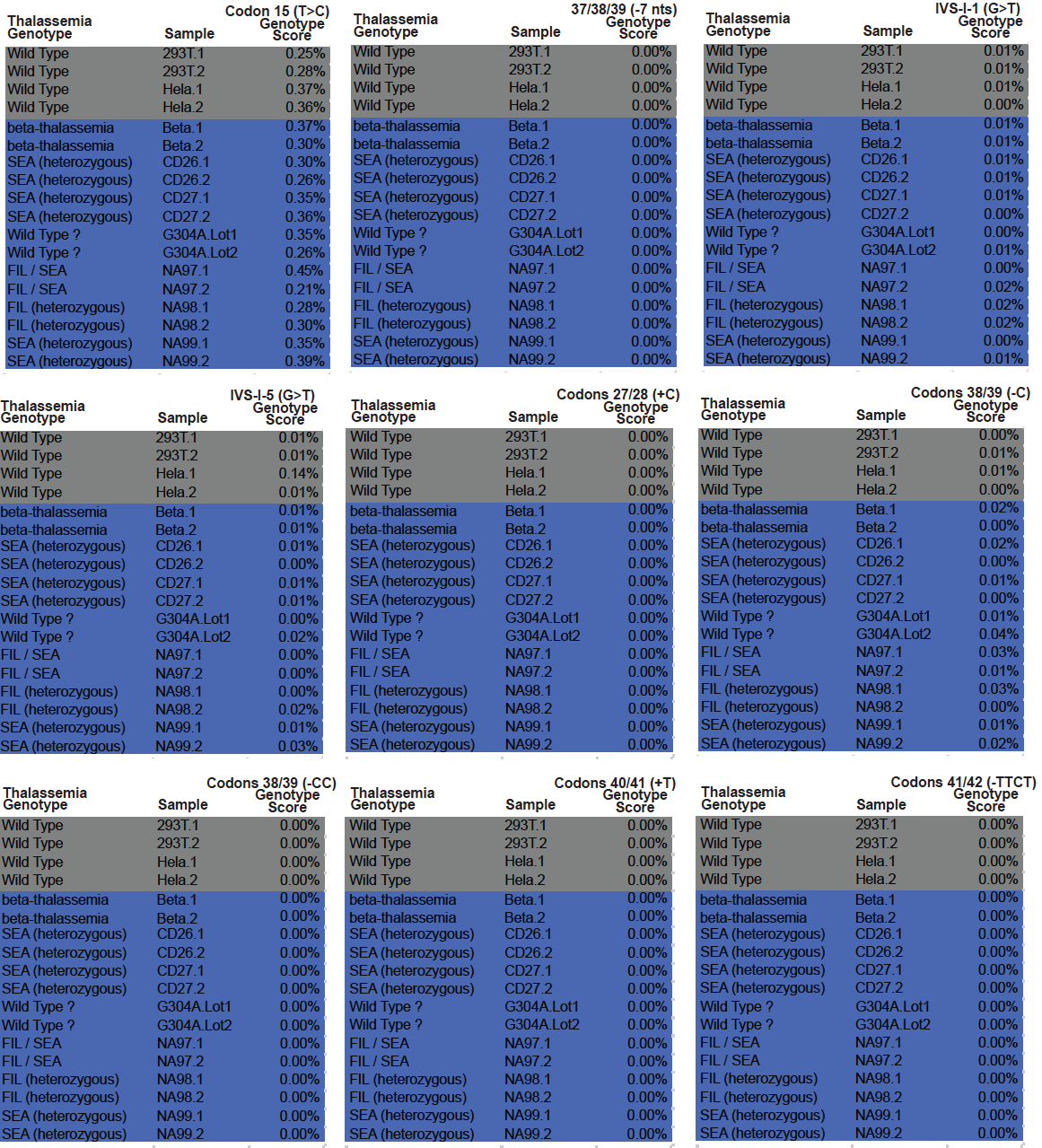


**Supplementary Figure S4.** Genotype scores of β-thalassemia mutations.


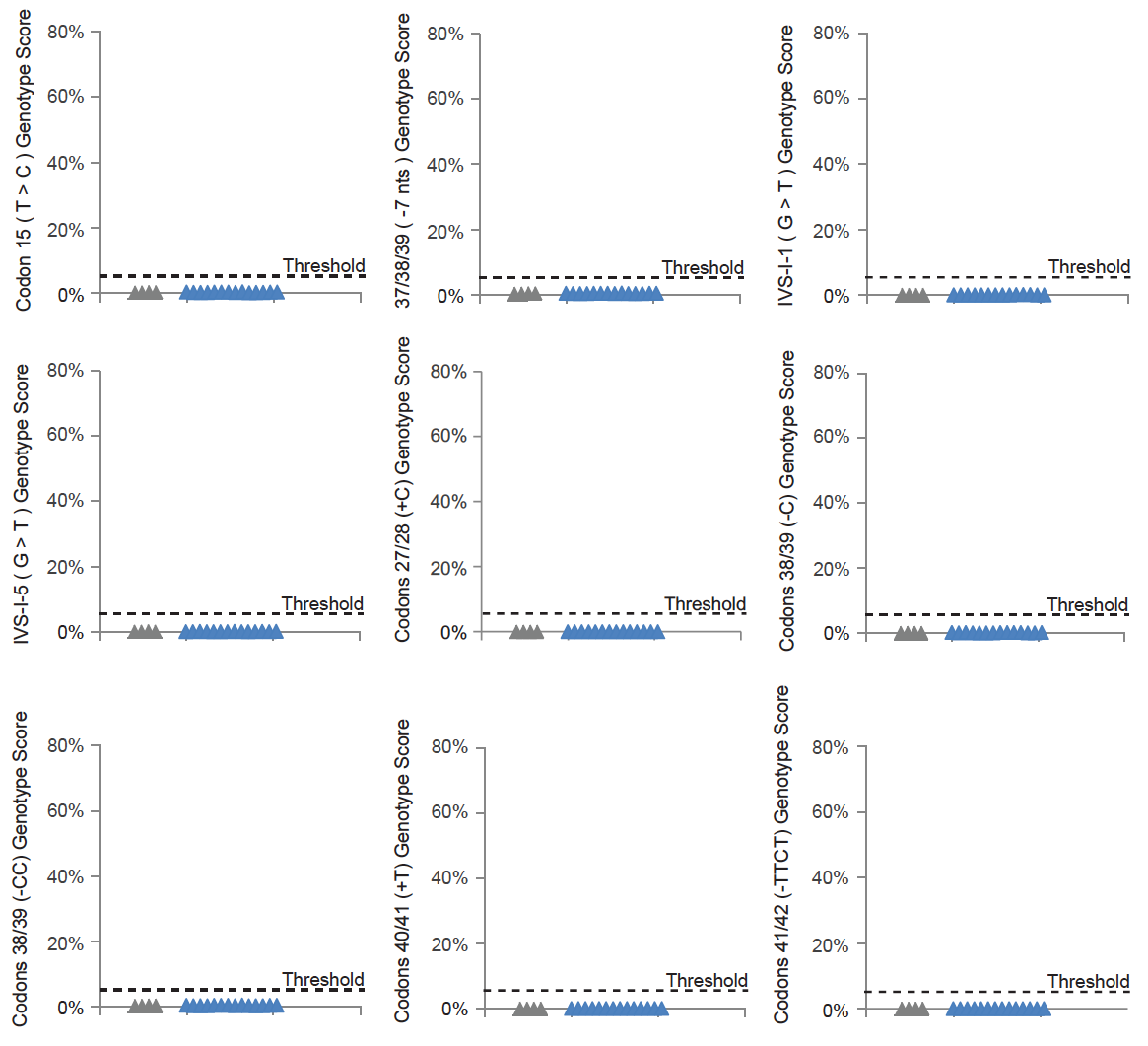


**Supplementary Figure S5.** Genotype calls of β-thalassemia mutations. Samples are labelled in grey (wild type) and blue (genotypes to be tested). Since all samples are negative for all the β-thalassemia mutations included in the figure, sample identities are not provided.


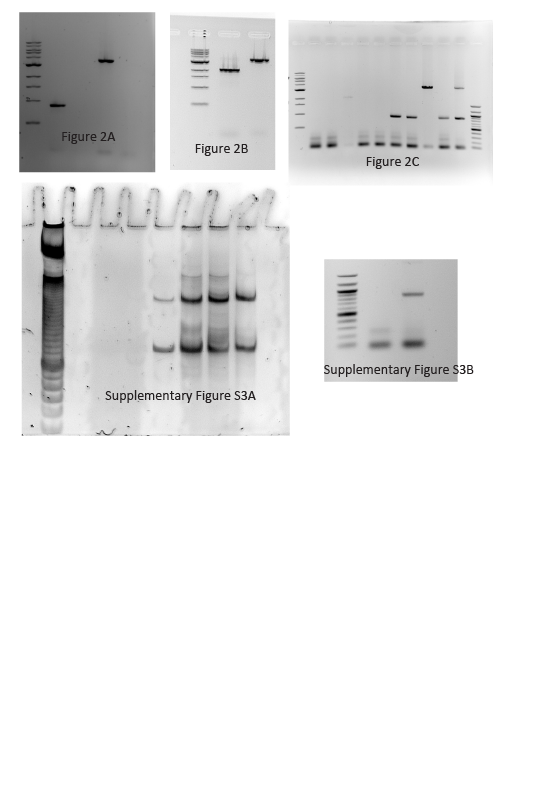


**Supplementary Figure S6.** Uncropped gel pictures of all the gels in the main text and in the supplementary materials.
